# *Lactobacillus delbrueckii*: A Functional Powerhouse in Dairy Fermentation and Emerging Probiotic Applications

**DOI:** 10.1002/fsn3.71546

**Published:** 2026-02-17

**Authors:** Yousef Nami, Anahita Barghi, Mahsa Sadeghi, Tara Farhadi, Babak Haghshenas

**Affiliations:** ^1^ Department of Food Biotechnology, Branch for Northwest and West Region, Agricultural Biotechnology Research Institute of Iran Agricultural Research, Education and Extension Organization (AREEO) Tabriz Iran; ^2^ Institute of Agricultural Life Science Dong‐A University Busan South Korea; ^3^ Students Research Committee Kermanshah University of Medical Sciences Kermanshah Iran; ^4^ Regenerative Medicine Research Center (RMRC), Health Technology Institute Kermanshah University of Medical Sciences Kermanshah Iran

**Keywords:** bioactive peptides (BAPs), exopolysaccharides (EPS), functional dairy foods, precision fermentation, strain specificity

## Abstract

*Lactobacillus delbrueckii* is a key lactic acid bacterium (LAB) widely used in dairy fermentation, particularly in the production of yogurt, cheese, and kefir. Its metabolic activities—such as efficient lactose utilization, rapid acidification, and high proteolytic activity—make it an indispensable starter culture in the dairy industry. Beyond its technological advantages, *L. delbrueckii* contributes significantly to human health through the production of bioactive peptides (BAPs), extracellular polysaccharides (EPS), and other functional metabolites with antioxidant, anti‐inflammatory, and immunomodulatory effects. Recent studies have also highlighted its role in gut health, cholesterol reduction, and potential anti‐cancer activity, underscoring its value as a probiotic. This review provides a comprehensive overview of the taxonomy, metabolism, and health‐promoting properties of *L. delbrueckii* while also exploring its potential applications in precision fermentation and the development of functional foods. The insights discussed herein position *L. delbrueckii* as a promising candidate for next‐generation probiotic dairy innovations.

## Graphical abstract

*Lactobacillus delbrueckii* is a key lactic acid bacterium widely used in dairy fermentation, particularly in yogurt and cheese production. This graphical abstract highlights its technological roles in lactose metabolism, acidification, proteolysis, and exopolysaccharide (EPS) production, which contribute to improved texture, flavor, and nutritional quality of fermented dairy products. In addition, fermentation‐derived metabolites, including bioactive peptides and EPS, are associated with gut health, immune modulation, and emerging functional food applications.

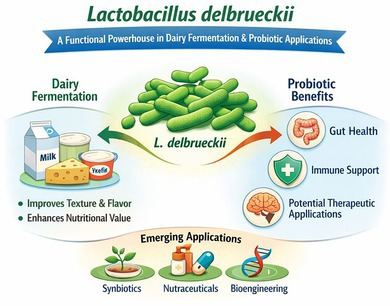

## 1 Introduction

Probiotics, defined as live microorganisms that confer health benefits when administered in sufficient amounts, have become central to modern nutrition and the development of functional foods. Many probiotic strains are naturally found in the gastrointestinal tract and other human‐associated niches, while others are administered through supplements and fermented foods to support gastrointestinal health, immunity, and metabolic function (Fijan 2023). Among probiotics, lactic acid bacteria (LAB) are commonly used in food microbiology as a functional and ecological group of mainly Gram‐positive, non‐spore‐forming bacteria that produce lactic acid as a major end‐product of the fermentation of carbohydrates, primarily simple sugars such as glucose and milk‐derived lactose, with substrate utilization patterns varying among species and ecological niches. Importantly, LAB do not represent a formal taxonomic rank and instead comprise multiple genera (e.g., Lactobacillus, Lactococcus, Leuconostoc, Pediococcus, Streptococcus, and others) that share convergent metabolic traits and food‐associated ecological niches (Gao et al. 2021).

Within LAB, the genus *Lactobacillus* contains over 260 species, including *Lactobacillus delbrueckii* , which is of particular interest in the dairy industry due to its functional and probiotic properties. Subspecies such as *L. delbrueckii* subsp. *bulgaricus* and *L. delbrueckii* subsp. *lactis* are widely used as starter cultures in yogurt, cheese, and kefir production (El Kafsi et al. 2014). Beyond fermentation, *L. delbrueckii* is recognized for its health‐promoting effects, including immune modulation, anti‐inflammatory activity, and enhanced lactose digestion through the secretion of bioactive peptides and extracellular polysaccharides (Bibi et al. 2021).

As interest in functional foods and precision fermentation grows, *L. delbrueckii* is increasingly studied for its potential to develop next‐generation probiotic products (De Jesus, Aburjaile, et al. 2022). Several reviews published in recent years have discussed probiotics and/or lactobacilli in a broad context, including a 2024 review that summarizes general trends and translational challenges for next‐generation probiotics (Abouelela and Helmy 2024).

However, despite the growing body of literature, key gaps remain for *L. delbrueckii* . Existing reports often address (i) dairy technological functions and (ii) health‐related outcomes in a fragmented manner, and many summaries do not explicitly grade the strength of evidence (in vitro vs. animal vs. human) at the strain level. In addition, rapid developments in *Lactobacillus* systematics and strain characterization warrant an updated, integrated synthesis that connects fermentation‐derived metabolites to host‐relevant mechanisms and clinically meaningful endpoints.

The distinct contribution of the present review is that it provides a species‐centered synthesis focused on *L. delbrueckii* , bridging both dairy fermentation technology and health‐related evidence. Specifically, we (i) contextualize the updated taxonomy/phylogeny and strain diversity of *L. delbrueckii* , (ii) summarize its technological roles across major dairy matrices and emerging precision fermentation applications, (iii) integrate mechanistic pathways linking fermentation outputs (e.g., proteolysis‐derived BAPs and EPS) to host‐relevant functions, and (iv) explicitly distinguish evidence from in vitro, animal, and human studies to avoid overinterpretation. This structured, evidence‐aware perspective is intended to support more rigorous strain selection and future translational research in functional dairy and beyond.

Expected outcomes of this review include (i) an evidence‐aware framework summarizing established versus preliminary effects of *L. delbrueckii* , (ii) identification of methodological and translational gaps (e.g., strain specificity, dose, matrix effects, and limited clinical trials), and (iii) guidance for future research and functional dairy development. This review aims to explore the taxonomy, metabolic pathways, health implications, and current and future applications of *L. delbrueckii* in dairy fermentation, with a particular focus on its relevance to human health and food biotechnology.

To improve readability, the review is organized as follows: Section 2 summarizes taxonomy and strain diversity; Sections 3 and 4 focus on core technological traits (fermentation performance and lactose catabolism); Section 5 synthesizes major bioactive properties and fermentation‐derived metabolites (BAPs, EPS, antimicrobial and emerging immune/allergy‐related bioactivities); Section 6 summarizes key dairy applications; Section 7 integrates probiotic mechanisms and health‐related evidence with explicit grading of in vitro/animal/human data; and Sections 8 and 9 discuss future perspectives and conclusions.

## 2 Examination of Taxonomy and Biodiversity

LAB are Gram‐positive, non‐spore‐forming microorganisms that convert glucose into lactic acid through fermentation. They belong to the phylum Bacillota (Firmicutes), class Bacilli, and order Lactobacillales, which comprises genera such as *Aerococcus*, *Enterococcus*, *Lactobacillus*, *Lactococcus*, *Leuconostoc*, *Pediococcus*, *Streptococcus*, and others (Guan et al. 2021). While LAB share functional characteristics, they do not constitute a monophyletic taxonomic group and exhibit considerable biodiversity. This diversity is reflected in their presence across varied ecological niches, including dairy products, meat, vegetables, soil, water, and the gastrointestinal tracts of humans and animals (Georgalaki et al. 2021).

**FIGURE 1:**
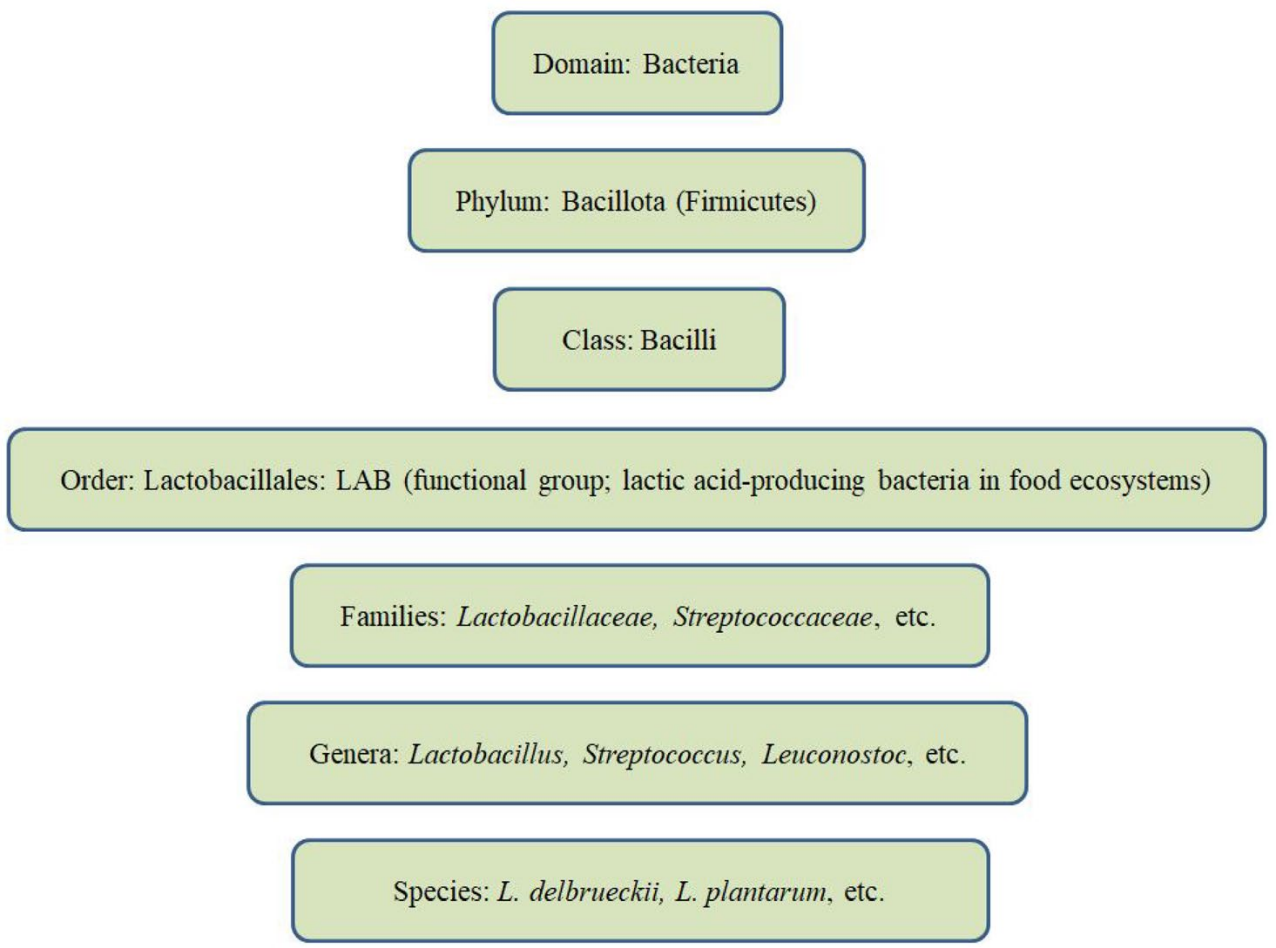
Phylogenetic position of *L. delbrueckii* within bacterial taxonomy shown as a simplified tree‐like scheme (phylum to species). The term LAB is indicated as a functional/ecological group commonly associated with food fermentations and characterized by lactic acid production, rather than a formal taxonomic rank.

The genus *Lactobacillus* consists of Gram‐positive, rod‐shaped, non‐spore‐forming bacteria commonly found in nutrient‐rich, carbohydrate‐dense environments. Before its reclassification in 2020, the genus included over 260 phylogenetically diverse species. A major taxonomic revision in 2020 reclassified the former genus *Lactobacillus* into multiple genera (e.g., *Lacticaseibacillus*, *Lactiplantibacillus*, *Limosilactobacillus*, and *Levilactobacillus*), while *Lactobacillus delbrueckii* remained within the emended genus *Lactobacillus*. In this review, the term LAB is used phenotypically/functional and taxonomic relationships are described using standard phylogenetic ranks (order/family/genus/species). Notably, *Lactobacillus delbrueckii* remains within the redefined genus *Lactobacillus*, classified under Group I of the *L. delbrueckii* cluster. Figure 1 provides a visual summary of the taxonomic hierarchy and diversity of LAB, highlighting the placement of *L. delbrueckii* .

*L. delbrueckii* is a facultative anaerobe that plays a significant role in dairy fermentations, particularly in the production of yogurt and cheese. It includes several subspecies, with *L. delbrueckii* subsp. *bulgaricus* and *lactis* being the most widely used starter cultures (El Kafsi et al. 2014). Studies, such as those by Song et al., have demonstrated genetic variability among *L. delbrueckii* subsp. *bulgaricus* strains, identifying distinct clonal complexes and region‐specific distribution patterns in fermented dairy products (Z. Song et al. 2024).

Recent genomic analyses by De Jesus et al. of multiple probiotic *L. delbrueckii* strains have revealed key genes associated with stress resistance, bile tolerance, surface‐layer proteins, antimicrobial compounds, and proteolytic activity (De Jesus, Aburjaile, et al. 2022). These functional traits may explain the probiotic potential of various *L. delbrueckii* strains used in food fermentation. Table 1 summarizes the functional capabilities of representative strains isolated from milk and dairy‐based products. This high degree of genetic and functional diversity within *L. delbrueckii* is crucial not only for understanding its ecological success but also for developing targeted starter cultures with specific technological and probiotic benefits in the dairy industry.

**TABLE 1:** Selected technological, survival, and other key functional characteristics of *L. delbrueckii* strains isolated from milk and milk products.

Strain	Property category	Specific property/characteristic	References
*L. bulgaricus* IMAU20312	Technological (fermentation)	Excellent fermentation properties: lactic acid production, viscosity enhancement, reduced syneresis, and flavor production during milk fermentation	(Dan et al. 2023)
*L. bulgaricus* LDB‐C1	Technological (EPS production and starter trait)	High EPS yield and good fermentation performance; contains CRISPR spacers (potential phage/plasmid resistance)	(Guan et al. 2021)
*L. delbrueckii* TUA4408L	Technological (fermentation)	Ability to grow and ferment soymilk	(Suda et al. 2021)
*L. bulgaricus* CICC 6047	Technological and bioactive compound production	Good milk growth; high acidification; produces EPS, GABA, folate, B‐vitamins, bacteriocins, and antioxidative compounds	(Song et al. 2024)
*L. bulgaricus* SRFM‐1	Technological (EPS production)	Produces r‐EPS suitable for functional foods (e.g., potential prebiotic)	(W. Tang et al. 2020)
*L. delbrueckii* GRIPUMSK	Bioactive compound production	Produces EPS with broad‐range antimicrobial activity	(Srinivash, Krishnamoorthi, Mahalingam, and Malaikozhundan 2023)
*L. delbrueckii* DMLD‐H1	Survival and technological trait	Bile and acid resistance; self‐cohesion; candidate for dairy fermentation	(J. Tang et al. 2023)
*L. delbrueckii* subsp. *lactis* CIDCA 133	Safety and probiotic trait	Resistant to aminoglycosides; no hemolysis or mucin degradation	(de Jesus, de Jesus Sousa, et al. 2022)
*L. delbrueckii* subsp. *bulgaricus* F17	Technological (biopreservation)	Biopreservative potential; reduces strawberry spoilage	(Fang et al. 2019)
*L. delbrueckii* subsp. *bulgaricus* ND02	Technological (processing)	Moderate acidity, high viscosity, good water holding; used in starter cultures	(Shao et al. 2014)
*L. delbrueckii* QS306	Bioactive compound production	Produces ACE‐inhibitory peptides during milk fermentation	(Wu et al. 2022)
*L. delbrueckii* subsp. *lactis* strain 313	Technological and bioactive production	Used in B_12_ assays; produces H_2_O_2_; acid‐tolerant, suitable for sour bread	(Agyei and Danquah 2012)
*L. delbrueckii* subsp. *bulgaricus* CRL 656	Technological (proteolysis)	Proteolytic action on I^2^‐lactoglobulin (may reduce antigenicity)	(Pescuma et al. 2011)

## 3 Technological Advantages of *L. delbrueckii*

*L. delbrueckii* is widely recognized as a key dairy starter due to a combination of technological traits that support efficient fermentation and desirable product quality. From an industrial perspective, its value lies not only in robust lactic acid production, but also in strain‐dependent capabilities related to lactose utilization, proteolysis, and exopolysaccharide (EPS) formation, which collectively influence acidification performance, texture, flavor development, and overall sensory acceptance in fermented dairy products. To reflect recent advances, this section and the subsequent technology‐focused sections prioritize original research evidence from the last decade (≈2015–2025), complemented by a limited number of foundational references where needed (Harlé et al. 2024).

A concise evaluation of the major technological advantages of *L. delbrueckii* can be summarized as follows. First, many strains exhibit strong lactose catabolism and acidification capacity, enabling rapid pH reduction and improved microbial safety and shelf stability in dairy matrices. Recent strain‐ and process‐level studies demonstrate that starter composition and strain ratios involving *L. delbrueckii* can measurably modulate fermentation kinetics, acidity development, and product quality attributes (e.g., viscosity and water‐holding capacity) in yogurt‐type systems (Dan et al. 2023).

Second, *L. delbrueckii* possesses a proteolytic system that supports growth in milk by liberating peptides and amino acids from caseins; these reactions are directly relevant to flavor formation and may also provide a mechanistic bridge to fermentation‐derived bioactive peptides discussed later in this review. Recent multi‐omics and genomic studies have further clarified how nitrogen acquisition and proteolysis‐related functions support rapid acidification and aroma‐relevant metabolite formation under dairy‐like conditions (Harlé et al. 2024).

Third, EPS‐producing strains can improve rheological properties such as viscosity and water‐holding capacity, contributing to texture enhancement and reduced syneresis, particularly in yogurt‐type products.

While these technological characteristics are well recognized, their magnitude is often strain‐specific and influenced by processing parameters (e.g., temperature, inoculum level, and co‐culture composition). Therefore, the following sections provide a more detailed discussion of lactose catabolism, proteolysis/BAP generation, and EPS production as distinct yet interconnected technological modules, aiming to present a structured basis for strain selection and functional dairy development.

## 4 Lactose Catabolism Potential of *L. delbrueckii*

**FIGURE 2:**
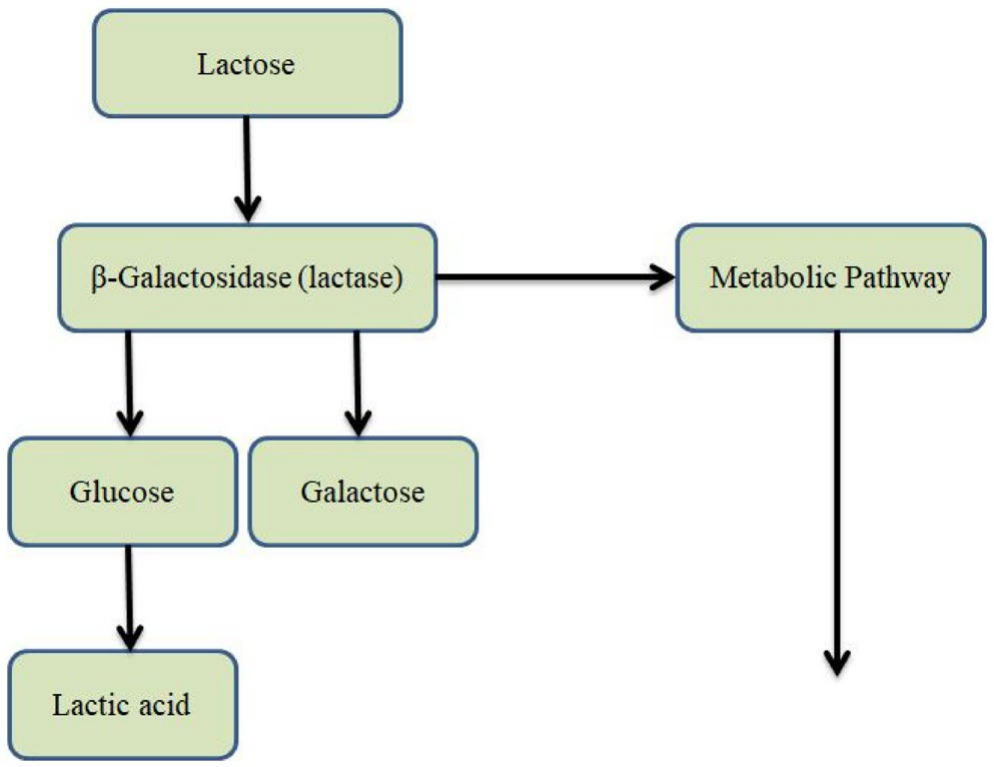
Metabolic pathway illustrating the breakdown of lactose by *L. delbrueckii* and subsequent production of lactic acid.

Lactose catabolism refers to the metabolic breakdown of lactose, a disaccharide composed of glucose and galactose linked via a *β* − 1 → 4 glycosidic bond, which constitutes 2%–8% of milk by weight. Hydrolysis of lactose is catalyzed by β‐galactosidase (lactase), producing monosaccharides that enter metabolic pathways such as glycolysis (for glucose) and the Leloir pathway (for galactose). This process is essential for nutrient utilization in mammals and forms the biochemical foundation for industrial dairy fermentations (Figure 2) (Greenwood‐Van Meerveld et al. 2017).

In line with recent original research, lactose conversion by *L. delbrueckii* has also been studied from an applied bioprocess perspective, where β‐galactosidase activity can contribute both to lactose hydrolysis and, in some strains/conditions, to the formation of galactooligosaccharides (GOS) via transgalactosylation—an industrially relevant trait for developing fermented dairy products with improved digestibility and potential prebiotic value (Arsov et al. 2022).

LAB, including *L. delbrueckii* , is well‐adapted to utilize lactose as a primary energy source. Through their robust lactose metabolism, these bacteria convert lactose into lactic acid under anaerobic conditions, thereby lowering the pH and enhancing the preservation, texture, and flavor of fermented dairy products such as yogurt and cheese. This catabolic capacity also makes lactose more digestible for individuals with lactose intolerance (Ibrahim et al. 2021).

*L. delbrueckii* employs homofermentative and heterofermentative pathways depending on the strain and substrate availability. In the homofermentative route, lactose is hydrolyzed and fermented predominantly into lactic acid. In contrast, heterofermentative strains may also produce carbon dioxide, acetate, or ethanol. Enzymes such as β‐galactosidase and lactate dehydrogenase play a key role in driving these fermentative processes (Giacon et al. 2022).

**FIGURE 3:**
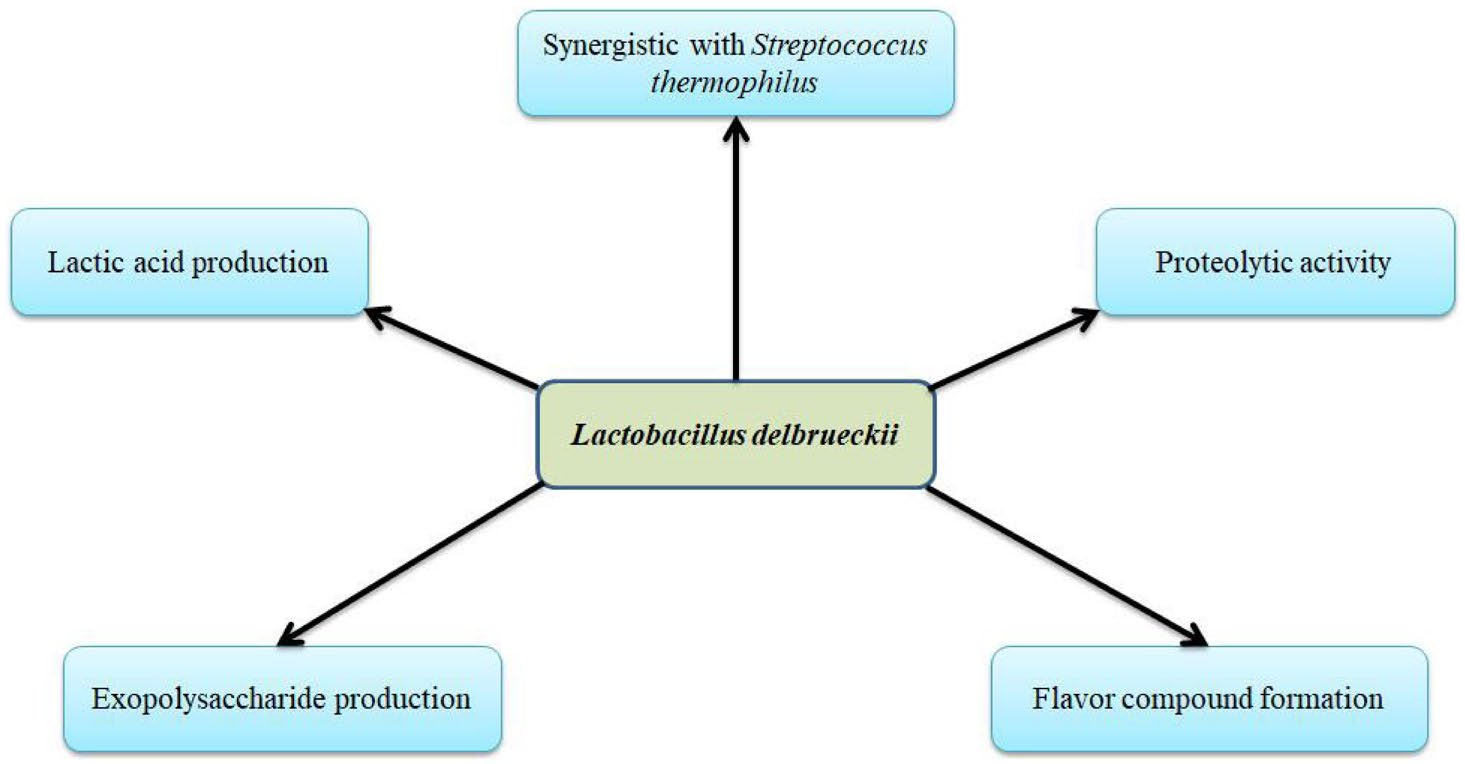
Functional and technological roles of *L. delbrueckii* in dairy fermentation, including lactose metabolism, lactic acid production, EPS synthesis, and proteolytic activity.

Notably, *L. delbrueckii* subsp. *bulgaricus* demonstrates a high affinity for milk environments due to horizontally acquired genes that enhance lactose uptake and metabolism. Proteomic and genomic investigations have provided strain‐level evidence for milk adaptation, highlighting metabolic streamlining and functional specialization consistent with strong performance in dairy fermentation (Yin et al. 2017). These genomic adaptations have enabled the bacterium to thrive in dairy niches, contributing significantly to fermentation efficiency and sensory quality. Comparative genomic analyses have shown that evolutionary pressures have streamlined its metabolism for optimal lactose utilization in milk‐based substrates (El Kafsi et al. 2014). Figure 3 illustrates the key functional and metabolic roles of *L. delbrueckii* in dairy fermentation. The catabolic versatility of *L. delbrueckii* , particularly its ability to rapidly ferment lactose, underpins its industrial relevance. This characteristic is central not only to its role in flavor and texture development but also to its probiotic value through improved digestibility and potential prebiotic interactions in the gut (Gao et al. 2021).

## 5 Bioactive Properties of *L. delbrueckii* and Fermentation‐Derived Metabolites

### 5.1 Overview: Bioactive Components and Evidence Considerations

*L. delbrueckii* can contribute to bioactivity in fermented dairy not only through viable cells but also through fermentation‐derived metabolites and cell‐associated components (often discussed under the broader concept of postbiotics). In dairy matrices, the major bioactive outputs most frequently reported for *L. delbrueckii* include proteolysis‐derived bioactive peptides (BAPs) and exopolysaccharides (EPS), alongside other antimicrobial factors (e.g., organic acids and, in some strains, bacteriocin‐like compounds). Importantly, reported bioactivities are typically strain‐dependent and are strongly influenced by processing parameters (e.g., temperature, inoculum level, fermentation time, and co‐culture composition) and by the food matrix. Therefore, this section organizes the main bioactive properties of *L. delbrueckii* into clearly defined subsections, while emphasizing that bioactivity claims should be interpreted according to evidence type (in vitro/animal/human), dose, and relevance of endpoints (Song et al. 2024; Chourasia et al. 2023).

### 5.2 Proteolytic System and Bioactive Peptide (BAP) Production

Proteolysis—the enzymatic hydrolysis of proteins into peptides and free amino acids—is fundamental to the growth and metabolism of lactic acid bacteria in milk‐based matrices and contributes to both the sensory attributes and nutritional value of fermented products (Song et al. 2024). In *L. delbrueckii* , proteolytic activity is particularly important because milk is a protein‐rich but relatively free‐amino‐acid–limited environment. Accordingly, *L. delbrueckii* relies on a coordinated proteolytic system to access nitrogen sources, while simultaneously generating peptide intermediates that may include BAPs with potential physiological functions (Table 2).

**TABLE 2:** Reported functional peptides produced by *L. delbrueckii* in milk and milk products.

Proteins	Peptides	Bioactivities	References
α_S1_‐casein	FVAPFPEVF SDIPNPIGSENSEK SDIPNPIGSEN FSDIPNPIGSEN RPKHPIKH	ACE‐inhibitory Antimicrobial Antioxidant, Antimicrobial Antioxidant ACE‐inhibitory	(Yu et al. 2021) (Yu et al. 2021) (Rubak et al. 2021) (Rubak et al. 2021) (Papadimitriou et al. 2007)
α_S2_‐casein	FTKKTKLTEEEKNRLN GPIVLNPWDQVK YQKA	ACE‐inhibitory ACE‐inhibitory ACE‐inhibitory	(Rubak et al. 2021) (Rubak et al. 2021) (Papadimitriou et al. 2007)
β‐casein	RELEELNVPGEIVESLSSSEESITR VYPFPGPIPN NIPPLTQTPV VENLHLPLPLL YQEPVLGPVRGPFPI LLYQEPVLGPVRGPFPIIV YQEPVLGPVRGPFPIIV	Antiproliferation ACE‐inhibitory ACE‐inhibitory ACE‐inhibitory Antimicrobial ACE‐inhibitory ACE‐inhibitory, Antimicrobial, Antithrombin, and Immunomodulatory	(Yu et al. 2021)
β‐casein	QEPVLGPVRGPFPIIV EPVLGPVRGPFP LNVPGEIVE NIPPLTQTPV QEPVLGPVRGPFP LGPVRGPFP DELQDKIHPF QEPVLGPVRGPFP VLGPVRGPFPII VVVPPFLQP LGPVRGPFP EPVLGPVRGPFP	ACE‐inhibitory ACE‐inhibitory ACE‐inhibitory ACE‐inhibitory ACE‐inhibitory ACE‐inhibitory ACE inhibitory ACE inhibitory Antimicrobial Antimicrobial ACE inhibitory Antioxidant, ACE inhibitory	(Yu et al. 2021) (Yu et al. 2021) (Gobbetti et al. 2000) (Gobbetti et al. 2000) (Villegas et al. 2014) (Villegas et al. 2014) (Rubak et al. 2021) (Rubak et al. 2021) (Rubak et al. 2021) (Rubak et al. 2021) (Rubak et al. 2021)
ĸ‐casein	ARHPHPHLSF	Antioxidant	(Rubak et al. 2021)
κ‐casein	LPYPY	Angiotensin‐converting enzyme inhibitory activity (C5)	(Wu et al. 2019)

The proteolytic system of *L. delbrueckii* typically comprises (i) cell‐envelope–associated proteinases that initiate casein hydrolysis, (ii) peptide transport systems that internalize oligopeptides, and (iii) intracellular peptidases that further process peptides to free amino acids required for cellular growth. Beyond supporting biomass formation and fermentation performance, this enzymatic cascade can release BAPs—short amino acid sequences that may exert biological effects such as angiotensin‐converting enzyme (ACE) inhibition, antioxidant activity, antimicrobial effects, and immunomodulatory actions (Brown et al. 2017; Chourasia et al. 2023). However, the occurrence and intensity of these activities are highly dependent on peptide sequence, concentration, and the surrounding food matrix, and thus should be interpreted in a strain‐ and context‐specific manner.

Importantly, the proteolytic capacity of *L. delbrueckii* varies across strains and subspecies due to genetic differences influencing proteinase expression and activity. A recent comparative genomic analysis of multiple *L. delbrueckii* genomes systematically characterized the diversity of key proteolytic components (including cell‐envelope proteinase features), reinforcing that proteolysis‐related potential is not uniform across strains (Elean et al. 2023). Strain‐level variation has practical relevance for dairy manufacturing because high‐proteolytic strains can enhance flavor development and texture through controlled casein breakdown, and may also increase the diversity of fermentation‐derived peptides of potential functional interest (Elean et al. 2023). For example, proteinases such as PrtB and related enzymes contribute to peptide release during fermentation, and recent genomic and peptidomic studies have reported strain‐specific repertoires of protease‐ and peptide‐associated genes and products (Ballini et al. 2023; Elean et al. 2023). One example frequently discussed in the literature is *L. delbrueckii* subsp. bulgaricus DQHXNS8L6, which has been reported to generate multiple peptides detectable both before and after simulated digestion, supporting its potential utility in functional dairy development (Elean et al. 2023).

Overall, proteolysis and BAP generation represent a key mechanistic bridge between the technological role of *L. delbrueckii* in dairy fermentation and its proposed health‐related applications. Future studies combining standardized fermentation conditions with robust peptide identification, quantification, and bioactivity validation—particularly in human‐relevant models—will be essential to clarify which strains and processing parameters reliably enhance beneficial peptide profiles. In parallel, fermentation optimization (and, where appropriate, targeted strain improvement) may support the development of value‐added fermented dairy products and nutraceutical concepts based on *L. delbrueckii* (Chourasia et al. 2023).

### 5.3 Exopolysaccharides (EPS) as Bioactive and Technological Metabolites

Extracellular polysaccharides (EPS) are high‐molecular‐weight carbohydrate polymers secreted by various microorganisms, including LAB. These biopolymers play a critical role in microbial ecology, particularly in biofilm formation, stress tolerance, and cell adhesion. In the context of food systems, EPS contributes significantly to the rheological and textural properties of fermented products, making them valuable functional components in dairy fermentation (Jurášková et al. 2022).

*L. delbrueckii* is known to produce strain‐specific EPS with varying structural and functional properties. These EPS not only enhance the viscosity, mouthfeel, and stability of fermented dairy products such as yogurt and buttermilk but also offer potential health benefits. Several studies have demonstrated that EPS derived from *L. delbrueckii* exhibit immunomodulatory, antioxidant, and anti‐inflammatory properties, supporting its classification as a postbiotic compound with biofunctional potential (Bibi et al. 2021).

EPS production in *L. delbrueckii* is influenced by environmental factors such as substrate composition, temperature, and the presence of specific inducers like inulin. Optimizing these conditions can increase EPS yield and enhance its functional properties (Jurášková et al. 2022). Original studies in fermented milk systems have also reported that EPS‐producing starter cultures can improve viscosity and water‐holding capacity and reduce syneresis, supporting the technological relevance of EPS for yogurt‐type products (Dan et al. 2023).

Furthermore, certain EPS‐producing strains of *L. delbrueckii* show improved survival in gastrointestinal conditions, suggesting a synergistic role in probiotic efficacy by enhancing mucosal adhesion and resistance to digestive stress (Penna et al. 2015). The dual role of EPS—as both a technological and bioactive agent—positions *L. delbrueckii* as a promising microorganism for the development of functional dairy foods. Future research on EPS biosynthesis pathways and structural characterization will enable the development of tailored applications in the food, nutraceutical, and pharmaceutical industries (Zhang et al. 2023).

### 5.4 Antimicrobial and Anti‐Biofilm Properties (Food‐Safety Relevance)

Beyond proteolysis‐derived peptides and EPS, *L. delbrueckii* can exert antimicrobial effects that are relevant to both food safety and host‐associated contexts. Antimicrobial activity may arise from multiple mechanisms, including organic acid production (environmental acidification), competition for nutrients and adhesion sites, and—in some strains—the secretion of bacteriocin‐like inhibitory substances and hydrogen peroxide. These activities can suppress or slow the growth of spoilage organisms and foodborne/pathogenic bacteria, supporting the use of *L. delbrueckii* as a protective culture in fermented dairy systems (Sadeghi et al. 2022; Huang et al. 2023).

Experimental studies have reported strain‐dependent inhibitory activity of *L. delbrueckii* against a range of clinically and food‐relevant bacteria, and some EPS fractions have also been explored for anti‐biofilm potential. However, the magnitude of inhibition is influenced by strain background, fermentation conditions, and the target organism, and in many cases evidence is derived from in vitro assays rather than validated in real food matrices or in vivo settings. Therefore, antimicrobial and anti‐biofilm properties should be considered promising but context‐dependent, requiring confirmation under standardized conditions and in application‐relevant models (Haghshenas et al. 2023; Nami et al. 2022).

### 5.5 Immunomodulatory Bioactivities and Relevance to Food Allergy (Emerging Evidence)

Immunomodulatory effects attributed to *L. delbrueckii* may involve both live‐cell interactions with intestinal immune components and the action of fermentation‐derived metabolites (postbiotic‐like effects), including peptides and EPS. Proposed mechanisms include modulation of cytokine profiles, enhancement of mucosal barrier integrity, and shifts in immune‐cell signaling that may support immune homeostasis. While several mechanistic studies suggest that specific strains can influence immune‐related endpoints, the evidence remains heterogeneous and often depends on experimental model, strain, dose, and matrix (Wu et al. 2022; Mizuno et al. 2020).

In the context of food allergy, which is increasingly recognized as a major issue in food safety and public health, emerging evidence indicates that LAB‐driven fermentation and selected microbial metabolites may reduce allergenicity of dairy proteins and influence tolerance‐related immune pathways. Studies have reported that fermentation‐associated metabolite profiles can reduce antigenicity of major whey proteins, supporting a potential processing‐based route for lowering exposure to allergenic epitopes. Additionally, preclinical work suggests that metabolites derived from *L. delbrueckii* (and related fermentation systems) may modulate immune responses in animal allergy models, consistent with improved tolerance. Nevertheless, current evidence for *L. delbrueckii* –specific allergy alleviation remains predominantly preclinical and indirect, and well‐designed human studies with standardized endpoints are required before firm clinical conclusions can be drawn. Accordingly, food allergy alleviation is best framed as an emerging and preliminary application of *L. delbrueckii* and its metabolites.

Overall, the bioactive profile of *L. delbrueckii* in dairy fermentation can be conceptualized as a modular output of (i) proteolysis‐derived peptides, (ii) EPS/postbiotic‐like polymers, and (iii) inhibitory metabolites that may support food safety and host‐relevant functions. While technological effects in dairy matrices are relatively well documented, many host‐directed bioactivities remain strain‐dependent and are supported predominantly by preclinical evidence. Accordingly, the subsequent health effects section (Section 7) emphasizes evidence grading and translational limitations to avoid overinterpretation.

## 6 Applications of *L. delbrueckii* in Different Food Product Groups

Fermentation performance and functional outcomes of *L. delbrueckii* are strongly influenced by the food matrix, processing conditions (e.g., temperature, fermentation time, oxygen exposure), and whether the organism is used as a starter (primary acidifier) or an adjunct/probiotic (functional add‐on). While *L. delbrueckii* is best established in thermophilic dairy fermentations, interest has expanded toward non‐dairy and hybrid products driven by consumer demand for functional foods, lactose‐free options, and sustainable protein sources. In practice, successful application requires aligning strain‐specific traits (acidification kinetics, proteolysis/BAP potential, EPS production, and stress tolerance) with product‐group constraints such as sugar availability, buffering capacity, texture formation, and sensory acceptance. The following subsections summarize applications by product group and provide a critical evaluation of current evidence and translational challenges.

### 6.1 Dairy Fermented Products

Dairy applications are supported by the most consistent technological evidence; however, health‐related claims still require strain‐specific clinical validation (Section 7).

#### 6.1.1 Yogurt and Yogurt‐Type Products

*L. delbrueckii* subsp. *bulgaricus* is a key thermophilic starter in yogurt fermentation, commonly used with *Streptococcus thermophilus* . Their symbiosis supports rapid acidification and contributes to characteristic flavor and texture. Depending on strain and process conditions, fermentation can also generate peptides and EPS that influence product quality (cross‐refer to Section 5) and may support digestive comfort in lactose‐sensitive consumers (cross‐refer to Section 7.1).

#### 6.1.2 Cheese (Hard/Semi‐Hard) and Ripened Dairy

*L. delbrueckii* subsp. *lactis* is frequently used as a thermophilic starter/adjunct in hard and semi‐hard cheeses, contributing to acidification and ripening‐associated proteolysis that shapes flavor development. The balance between desired proteolysis and avoidance of bitterness is strain‐ and process‐dependent.

#### 6.1.3 Buttermilk and Cultured Dairy Beverages

In cultured buttermilk‐type products, *L. delbrueckii* contributes to acidification and may influence viscosity/texture through EPS production (Section 5.3). Its use is typically optimized via co‐cultures and careful fermentation control.

#### 6.1.4 Kefir‐Like Beverages (Controlled/Industrial Formulations)

Although not always dominant in traditional kefir grains, *L. delbrueckii* can be added to defined starters for kefir‐like products to contribute acidification and sensory attributes, while overall functionality depends on the consortium design.

*L. delbrueckii* plays a pivotal role in the fermentation of various dairy products due to its ability to metabolize lactose, produce lactic acid, and contribute to the sensory and textural quality of the final product. Its primary subspecies— *L. delbrueckii* subsp. *bulgaricus* and *lactis*—are widely utilized in the dairy industry as starter cultures (El Kafsi et al. 2014).

##### 6.1.4.1 Yogurt

*L. delbrueckii* subsp. *bulgaricus*, often used in conjunction with *Streptococcus thermophilus* , is a key component of traditional yogurt fermentation. This symbiotic relationship accelerates acidification, enhances fermentation efficiency, and contributes to the characteristic tangy flavor and creamy texture of the product—moreover, the metabolic activities of *L. delbrueckii* subsp. *Bulgaricus* releases bioactive peptides and exopolysaccharides with potential health benefits (Gao et al. 2021).

##### 6.1.4.2 Cheese

*L. delbrueckii* subsp. *lactis* is commonly employed in cheese fermentation, particularly in hard and semi‐hard varieties such as Cheddar, Emmental, and Parmesan. It contributes to milk acidification, proteolysis during ripening, and the development of complex flavor compounds. Its metabolic profile enhances the elasticity, texture, and maturation characteristics of aged cheeses (Azzouz et al. 2024).

##### 6.1.4.3 Buttermilk

Cultured buttermilk is produced by fermenting low‐fat milk using *L. delbrueckii* subsp. bulgaricus, either alone or in combination with other LAB. The strain's ability to ferment lactose contributes to the tangy flavor and thick texture while also improving digestibility and adding nutritional value such as B vitamins and calcium (Pereira et al. 2024).

##### 6.1.4.4 Kefir

Though not a native species in traditional kefir grains, *L. delbrueckii* subsp. *bulgaricus* can be introduced into controlled kefir fermentations. It works synergistically with other LAB and yeasts to influence flavor, texture, and probiotic potential. The inclusion of *L. delbrueckii* has been linked to improved gut health and metabolic support (Dahiya and Nigam 2022).

In summary, the functional versatility of *L. delbrueckii* across diverse dairy matrices—ranging from yogurt to aged cheeses and fermented beverages—highlights its central role in industrial dairy fermentation. Its dual contributions to product quality and potential health benefits reinforce its importance as a target organism in the development of next‐generation probiotics and functional foods. Examples of *L. delbrueckii* strains used in the development of various fermented dairy products are presented in Table 3.

**TABLE 3:** Overview of *L. delbrueckii* strains used in the production of specific or functionally improved fermented dairy products.

Type of product	Strain(s)	Key contribution/effect on product or its functionality	References
Yogurt	*L. bulgaricus* IMAU20312	Affects yogurt consistency (e.g., improves viscosity, reduces syneresis)	(Dan et al. 2023)
Cheese (functionally studied)	*L. delbrueckii* CNRZ327	Used in cheese production, the resulting product was investigated for potential anti‐inflammatory properties (see Table 4 for host health effects)	(El Kafsi et al. 2014)
Fermented milk (functionally studied)	*L. delbrueckii* subsp. *bulgaricus* SRFM1	Contributes to fermented milk exhibiting enhanced antioxidant capacity (see Table 4 for potential host health effects)	(Tang et al. 2020)
Traditional Indian dairy (dahi type)	*L. delbrueckii* subsp. *indicus* NCC725	Characterizes Dahi by exclusive D‐lactic acid production, influencing product's final properties	(Dellaglio et al. 2005)
Pecorino cheese	*L. delbrueckii* P7, P9, P10, P11, P12, P13, P14, P15, P33, P36, P37, P39, P40	These strains contribute to flavor and texture development in Pecorino cheese, for example, via proteolytic activity, diacetyl production, and/or exopolysaccharide production	(Nicosia et al. 2023)
Dairy Products (General)	*L. delbrueckii* subsp. *bulgaricus* 76	Contributes to preservation and typical sensory characteristics through the production of organic acids and hydrogen peroxide	(Dellaglio et al. 2005)
Naturally fermented yak milk	*L. delbrueckii* subsp. *bulgaricus* strain ND02	Imparts desirable processing properties to the product, such as moderate acidity, high viscosity, and good water‐holding capacity	(Shao et al. 2014)
Pecorino del Reatino (Italian Cheese)	*L. delbrueckii* subsp. *bulgaricus* PR1	Results in cheese enriched with high concentrations of γ‐aminobutyric acid (GABA), a known functional compound	(Siragusa et al. 2007)
Yogurt	*L. delbrueckii* subsp. *bulgaricus* SIT‐17.B	Contributes to improving the overall flavor profile of yogurt	(Tian et al. 2024)

### 6.2 Application of *L. delbrueckii* in the Production of Yogurt

*L. delbrueckii* subsp. *bulgaricus* is a key lactic acid bacterium used as a starter culture in yogurt fermentation, typically in symbiotic association with *Streptococcus thermophilus* . This partnership is crucial for efficient lactose fermentation, which facilitates the rapid acidification of milk and contributes to the development of yogurt's characteristic flavor and texture (Dan et al. 2023). Their metabolic interactions also enhance the release of peptides and other compounds that improve both the sensory and nutritional qualities of the final product (McGrail 2022).

*L. delbrueckii* subsp. *bulgaricus* is particularly adapted to milk environments, having undergone reductive evolution that streamlined its genome for efficient lactose metabolism. In contrast, *L. delbrueckii* subsp. *lactis* retains a broader carbohydrate utilization capacity, attributed in part to horizontally acquired genes. These genomic adaptations have enabled each subspecies to specialize in distinct functional niches within dairy fermentation (El Kafsi et al. 2014).

Studies have demonstrated that yogurts fermented with *L. delbrueckii* may contribute to various health benefits, including improved gut health and immune modulation (as discussed in Section 9). These probiotic attributes, along with their technological roles, make the *L. delbrueckii* subspecies indispensable to yogurt production (Mirsalami and Mirsalami 2024). From both culinary and nutritional perspectives, yogurt remains a globally valued staple food. The contribution of *L. delbrueckii* —particularly subsp. *bulgaricus*—to its flavor, texture, and potential health benefits underscores its continued relevance in dairy biotechnology (Gao et al. 2021).

### 6.3 Application of *L. delbrueckii* in the Production of Cheese

*L. delbrueckii* subsp. *lactis* is widely utilized as a thermophilic adjunct or starter culture in the production of various hard and semi‐hard cheeses, such as Cheddar, Gouda, and Parmesan. Its primary roles in cheese‐making include promoting rapid acidification through lactose fermentation, contributing to the development of desired texture, and enhancing flavor profiles during ripening (Buchin et al. 2017).

During cheese maturation, *L. delbrueckii* exhibits significant proteolytic and lipolytic activity, breaking down caseins and milk fats into a wide range of peptides, amino acids, and volatile compounds. These metabolic products contribute to the complex sensory attributes of aged cheeses, including nutty, buttery, and savory notes. Its presence also helps reduce bitterness by releasing small peptides that modulate flavor (McSweeney 2004).

In particular, the thermal tolerance and metabolic flexibility of subsp. lactis make it suitable for high‐temperature cheese processes. It is often combined with other lactic acid bacteria to improve consistency and safety in industrial cheese production. Additionally, in traditional raw milk cheeses, naturally occurring populations of *L. delbrueckii* may influence the microbial ecology and final organoleptic properties of the product (Azzouz et al. 2024).

Whether employed as part of a defined starter culture or occurring spontaneously in artisanal processes, *L. delbrueckii* subsp. *lactis* plays an essential role in the quality, safety, and distinctiveness of a wide range of cheeses. Its contribution to flavor and texture, along with its probiotic potential, supports its ongoing application in both traditional and modern dairy technology (Antonsson et al. 2002).

### 6.4 Application of *L. delbrueckii* in the Production of Buttermilk

Buttermilk, traditionally defined as the liquid byproduct of butter churning, has evolved into a deliberately cultured dairy product produced through the fermentation of low‐fat milk by selected LAB. Among the key species used in cultured buttermilk production is *L. delbrueckii* , particularly subsp. *bulgaricus*, although subsp. lactis may also be employed (Pereira et al. 2024).

During fermentation, *L. delbrueckii* metabolizes lactose into lactic acid, resulting in the characteristic sour taste and decreased pH of buttermilk. It is often used in combination with *S. thermophilus* to initiate and sustain fermentation, reflecting the symbiotic interactions commonly utilized in dairy processing. The production of exopolysaccharides and proteolytic activity by *L. delbrueckii* also contributes to the texture, viscosity, and sensory appeal of the final product (Tarique 2024).

Buttermilk enriched with *L. delbrueckii* offers not only desirable technological properties but also nutritional and potential probiotic benefits. It is a natural source of calcium, potassium, and B‐complex vitamins, and its live cultures may support digestive health and improve gut microbiota balance (as discussed in Section 9) (Sahoo et al. 2023). In summary, *L. delbrueckii* plays a crucial role in modern buttermilk production, enhancing both the functional characteristics and health benefits of this traditional yet evolving dairy beverage (Kaur et al. 2022).

### 6.5 Application of *L. delbrueckii* in the Production of Kefir

Milk kefir is a fermented dairy beverage produced using kefir grains—complex symbiotic consortia of LAB, acetic acid bacteria, and yeasts. The microbial composition of kefir grains varies depending on geography, environmental conditions, and culturing practices. More than 50 bacterial species have been identified in kefir, including *Lactobacillus acidophilus* , *Lacticaseibacillus casei*, *Levilactobacillus brevis*, *S. thermophilus* , and occasionally *L. delbrueckii* subspecies *bulgaricus* and *lactic*. Yeast species commonly found in kefir include *Saccharomyces cerevisiae* , *Kluyveromyces lactis*, and various *Kazachstania* and *Candida* species (Leite et al. 2013).

While *L. delbrueckii* is not considered a dominant native species in traditional kefir grains, it can be introduced as part of a defined starter culture in industrial or controlled fermentations to produce kefir‐like beverages. In such settings, *L. delbrueckii* , particularly subsp. *bulgaricus*, contributes to lactose fermentation, lactic acid production, and textural enhancement, improving the viscosity and flavor of the final product (Dahiya and Nigam 2022).

The role of yeasts in kefir includes releasing B‐group vitamins and amino acids, which support LAB growth and fermentation performance. The distinctive tangy flavor of kefir is primarily attributed to lactic acid and other metabolic byproducts generated by LAB. When included in kefir formulations, *L. delbrueckii* may contribute to gut health and improve lactose digestion (as discussed in Section 9), thereby enhancing the probiotic potential of the beverage. Overall, the incorporation of *L. delbrueckii* into kefir production—especially in designed starter systems—can offer both functional and health‐related benefits, supporting its relevance in modern probiotic beverage development (W. Tang et al. 2020).

## 7 Probiotic Mechanisms and Health‐Related Effects of *L. delbrueckii*

Probiotics are live microorganisms that, when consumed in adequate amounts, confer health benefits to the host by modulating the gut microbiota, supporting immune function, and enhancing metabolic balance. LAB are widely used in functional foods due to their long history of safe use and diverse physiological activities (Fang et al. 2019).

*L. delbrueckii* , a species extensively applied in dairy fermentation, exhibits several probiotic‐relevant mechanisms, including adhesion to intestinal epithelial cells, competitive exclusion of pathogens, production of antimicrobial substances (e.g., organic acids and bacteriocins), modulation of cytokine responses, and support of gut barrier function. In addition, *L. delbrueckii* contributes to the formation of functional metabolites and fermentation‐derived compounds such as BAPs, EPS, and short chain fatty acids (SCFAs), which may collectively influence gastrointestinal and systemic physiology (Tang et al. 2023).

The health‐related outcomes attributed to *L. delbrueckii* are often strain‐dependent and influenced by dosage, intervention duration, food matrix, and host factors. Accordingly, this section integrates the major mechanisms and reported health effects while explicitly distinguishing evidence derived from in vitro studies, animal models, and human clinical investigations (Sanders et al. 2019; de Jesus, Santos, et al. 2024). An evidence‐graded overview of reported health effects is summarized in Table 4.

**TABLE 4:** Reported health effects of *L. delbrueckii* strains as probiotic bacteria.

Probiotic trait/mechanism leading to effect	Strain(s)	Health‐related probiotic effect(s)	References
Detoxification/protective effect	*L. delbrueckii* KLDS1.0207	Alleviation of lead (Pb) toxicity	(Li et al. 2017)
Anti‐inflammatory/mucosal protection	*L. delbrueckii* subsp. *lactis* CIDCA 133	Amelioration of 5‐FU‐induced intestinal mucositis (specific anti‐inflammatory/immunomodulatory effect)	(De Jesus et al. 2019)
Immunomodulation (via whole cell/components)	*L. delbrueckii* TUA4408L	Modulation of innate immune response of intestinal epithelial cells (in vitro evidence)	(Suda et al. 2021)
Metabolic regulation/psychobiotic effects	*L. bulgaricus* TCI904	Pancreatic lipase inhibition (in vivo); reduction in body weight gain; immune modulation; metabolic improvement; anxiolytic properties (in HFD‐induced obese mice)	(Lin et al. 2022)
Prebiotic effect (associated with its EPS)	*L. bulgaricus* SRFM‐1	Exopolysaccharides (EPS) produced by this strain exhibit potential prebiotic property beneficial for gut health	(W. Tang et al. 2020)
Peptide‐mediated bioactivity	*L. delbrueckii* QS306	Potential antihypertensive effect associated with Angiotensin‐Converting Enzyme (ACE) inhibitory peptides produced in fermented milk	(Wu et al. 2023)
Immunomodulation/anti‐fatigue (via product consumption)	*L. bulgaricus* OLL1073R‐1	Stimulation of the immune system; reduction in common cold risk; amelioration of summer heat fatigue (effects observed from yogurt consumption)	(Hemmi et al. 2023)
Reduction of allergenicity (via proteolysis)	*L. delbrueckii* subsp. *bulgaricus* CRL 656	Reduction of antigenic response to bovine β‐lactoglobulin through specific proteolytic action (potential for hypoallergenicity)	(Pescuma et al. 2011)
Antimicrobial/antioxidant/anti‐cancer potential (specific strain effects not covered by EPS/bile resistance entries)	*L. delbrueckii* GRIPUMSK	Direct antimicrobial and antioxidant effects; potential for cancer cell abatement (beyond EPS‐mediated effects if applicable)	(Srinivash, Krishnamoorthi, Mahalingam, Malaikozhundan, and Keerthivasan 2023)

### 7.1 Digestion and Absorption of Nutrients

Efficient digestion and nutrient absorption are essential to human health, enabling dietary macronutrients and micronutrients to become bioavailable. Probiotic microorganisms may support these processes through enzymatic activities, modulation of gut microbiota composition, and improvement of gastrointestinal function (Roberfroid et al. 2010).

In the context of fermented dairy foods, *L. delbrueckii* is most consistently linked to digestive comfort and improved lactose tolerance in individuals with lactase deficiency. As detailed earlier in the manuscript (Section 3), this benefit is primarily related to fermentation‐associated enzymatic activity and product characteristics; therefore, the present subsection focuses on application‐oriented evidence rather than reiterating metabolic pathways.

From a practical standpoint, clinical and population‐level observations suggest that consumption of yogurt/fermented milk containing *L. delbrueckii* (often in combination with other starter cultures) may reduce common lactose intolerance–related symptoms such as bloating, abdominal discomfort, and diarrhea, thereby improving overall tolerance to dairy intake. These outcomes are generally attributed to the food matrix and viable starter cultures delivered with the product, although the magnitude of benefit can vary depending on strain composition, dose, frequency of consumption, and host factors (De Jesus, Aburjaile, et al. 2022; Yeboah 2023).

Beyond lactose tolerance, some strains of *L. delbrueckii* can produce metabolites such as EPS that may help maintain a gut environment conducive to nutrient absorption. By supporting epithelial integrity and microbial balance, fermented dairy products containing *L. delbrueckii* may contribute indirectly to digestive efficiency, although these effects require more standardized human studies to determine consistency and strain specificity (Ballini et al. 2023). Overall, while mechanistic data are informative, the most actionable evidence at present supports *L. delbrueckii* –containing fermented dairy foods as a dietary approach to improving digestive tolerance in lactose‐sensitive populations.

### 7.2 Reducing Inflammation

Inflammation is a fundamental component of the immune response; however, chronic low‐grade inflammation is associated with metabolic and inflammatory disorders. The potential anti‐inflammatory effects of *L. delbrueckii* have been investigated across experimental systems, although the strength of evidence varies across in vitro, animal, and human studies (Calder et al. 2017).

Several in vitro studies suggest that selected strains may modulate inflammatory signaling pathways by influencing cytokine profiles in intestinal epithelial and immune cells. Reports include reduced expression of pro‐inflammatory mediators such as TNF‐α and IL‐6 alongside increases in anti‐inflammatory cytokines such as IL‐10 (Fang et al. 2019; Ballini et al. 2023).

Evidence from animal models further supports these observations. Administration of *L. delbrueckii* strains in rodent models of intestinal inflammation has been associated with attenuation of inflammatory markers, improvement of gut barrier integrity, and reduction of oxidative stress. These effects may involve microbiota interactions and SCFA‐related pathways (de Jesus, Santos, et al. 2024; Shah and Shim 2025).

In contrast, human clinical evidence remains limited and heterogeneous. Although consumption of fermented dairy products containing *L. delbrueckii* has been linked to improved immune balance or reduced inflammatory symptoms in some settings, outcomes appear strain‐specific and influenced by dose, duration, and host characteristics. Thus, anti‐inflammatory benefits in humans should currently be regarded as potential rather than established, supporting the need for larger, well‐controlled clinical trials (Rupa and Mine 2012).

### 7.3 Boosting the Immune System

The immune system plays a vital role in defense against pathogens and maintenance of homeostasis. Probiotics may modulate both innate and adaptive responses via interactions with the gut‐associated lymphoid tissue (GALT) (Suda et al. 2021).

*L. delbrueckii* has been studied for immunomodulatory effects, including modulation of cytokine production and interaction with dendritic cells and macrophages. These effects may contribute to immune homeostasis and reduced susceptibility to infections in a strain‐dependent manner (Wu et al. 2022).

In addition, *L. delbrueckii* has been associated with increased secretory IgA (sIgA) production and enhancement of certain immune functions such as NK cell activity. For example, improved antigen‐specific responses have been reported in studies evaluating immune outcomes after consumption of fermented dairy products in vaccination‐related contexts (Santiago‐López et al. 2015). Emerging work also suggests that EPS‐related immunomodulation may influence innate antiviral responses; however, more clinical evidence is needed to define effective strains, endpoints, and dose–response relationships (Mizuno et al. 2020). Beyond infection‐related outcomes, immunomodulation by *L. delbrueckii* has also been explored in the context of food allergy, where both live cells and fermentation‐derived metabolites may contribute to tolerance‐related immune pathways.

#### 7.3.1 Food Allergy Alleviation (Emerging Evidence)

Food allergy is an increasingly important concern in food safety and public health. As outlined in Section 5.5, emerging evidence suggests that fermentation with selected LAB and fermentation‐derived metabolites may reduce allergenicity of dairy proteins and influence tolerance‐related immune pathways. However, for *L. delbrueckii* specifically, available data remain predominantly preclinical and heterogeneous, and robust human trials with standardized clinical endpoints are still limited. Therefore, food allergy alleviation should be considered a preliminary and emerging application requiring further validation.

### 7.4 Reducing Cholesterol Levels

Hypercholesterolemia is a major risk factor for cardiovascular disease, and probiotics have been explored as complementary dietary strategies for lipid management. Some LAB—including selected *L. delbrueckii* strains—may influence cholesterol metabolism through multiple pathways (Nami et al. 2019).

One proposed mechanism involves bile salt hydrolase (BSH) activity, which can deconjugate bile salts, reduce reabsorption, and increase bile acid excretion, thereby promoting hepatic conversion of cholesterol into new bile acids (Begley et al. 2005). Additional mechanisms include cholesterol assimilation into bacterial membranes during growth and SCFA production (particularly propionate), which have been linked to reduced hepatic cholesterol synthesis (Yu et al. 2021).

Animal studies and small‐scale human studies suggest that fermented dairy products containing probiotics may improve lipid profiles, including reductions in total cholesterol and LDL cholesterol. However, effects are likely strain‐ and context‐dependent, and further well‐controlled human trials are required to clarify clinical relevance and magnitude of benefit (Ejtahed et al. 2011).

### 7.5 Eradication of *Helicobacter pylori* Infection

*Helicobacter pylori* colonizes the gastric mucosa and contributes to gastritis, peptic ulcer disease, and gastric cancer risk. While antibiotic therapy remains the standard of care, antibiotic resistance and adverse effects have stimulated interest in adjunct probiotic strategies (Goderska et al. 2018).

Experimental and clinical literature suggests that certain *L. delbrueckii* strains may inhibit *H. pylori* through competition for adhesion sites, production of organic acids, and secretion of antimicrobial compounds such as bacteriocins. Probiotics may also support mucosal barrier integrity and modulate inflammation (Juntarachot et al. 2023).

In clinical contexts, co‐administration of fermented milk or yogurt containing probiotic strains has been associated with improved eradication rates and reduced gastrointestinal adverse effects in some studies. Nonetheless, outcomes are strain‐specific and dose‐dependent, and additional randomized controlled trials are needed to standardize interventions and confirm therapeutic value (Penumetcha et al. 2021; Tanashat et al. 2024).

### 7.6 Antimicrobial Activity

Antimicrobial activity is one of the mechanisms through which probiotic strains may support host health and reduce pathogen burden. As discussed in Section 5.4, *L. delbrueckii* can inhibit undesirable microorganisms via acidification, competitive exclusion, and strain‐dependent inhibitory metabolites. In host‐related contexts, these effects may contribute to reduced pathogen colonization and improved microbial balance; however, most evidence remains strain‐specific and is frequently derived from in vitro assays. More studies in application‐relevant models and human settings are required to determine clinical significance and dose–response relationships (Huang et al. 2023; Haghshenas et al. 2023).

### 7.7 Gut Wellness

Gut wellness refers to gastrointestinal functionality, including efficient digestion, nutrient absorption, immune regulation, and protection against pathogens. A stable and diverse gut microbiota is central to these outcomes (Obayomi et al. 2024).

Fermented dairy products delivering probiotics may support gut wellness by maintaining microbial balance, inhibiting pathogen colonization, and strengthening the mucosal barrier. *L. delbrueckii* may contribute through competitive exclusion, enhancement of mucus layer integrity, and metabolite production (Dahiya and Nigam 2022).

Animal studies suggest that certain strains can influence microbiota composition and increase SCFA production, which may support barrier function and reduce inflammation. Encapsulation approaches have also been explored to improve survival under gastrointestinal stress and to mitigate dysbiosis in experimental settings (de Jesus, dos Santos Freitas, et al. 2024; L. Li et al. 2023; Nami et al. 2023). While preclinical results are encouraging, more human research is required to clarify strain‐specific contributions and optimize probiotic formulations.

### 7.8 Potential and Preliminary Applications of *L. delbrueckii*

In addition to established technological roles and more extensively studied probiotic effects, *L. delbrueckii* has been explored for several potential applications for which the current evidence remains preliminary. These emerging areas include gestational diabetes management, anti‐tumor activity, and psychobiotic effects. Available data are largely derived from experimental models, small‐scale studies, or indirect mechanistic evidence and should therefore be interpreted cautiously.

The role of *L. delbrueckii* in the prevention or management of gestational diabetes mellitus (GDM) is not yet well established. While probiotics broadly have shown mixed effects on glucose metabolism and insulin sensitivity during pregnancy, evidence specifically for *L. delbrueckii* is scarce. Many studies involve multi‐strain formulations, limiting attribution to this species. To date, no well‐designed randomized controlled trials have directly evaluated *L. delbrueckii* as a single‐species intervention for GDM outcomes (Lindsay et al. 2013; Mu et al. 2023).

Similarly, the anti‐tumor potential of *L. delbrueckii* has been investigated primarily in in vitro and animal studies. Proposed mechanisms include modulation of inflammation and oxidative stress, interaction with carcinogenic compounds, and indirect microbiota‐mediated effects. However, clinical evidence in humans is insufficient to support definitive preventive or therapeutic claims at present (Yu and Li 2016; Jampílek et al. 2022).

Interest in psychobiotic effects has also increased due to recognition of the gut–brain axis. Limited human studies have reported modest improvements in psychological well‐being or quality‐of‐life outcomes after consumption of yogurt fermented with specific strains, but findings are constrained by small sample sizes, heterogeneous endpoints, and strain‐specific effects. Accordingly, psychobiotic roles should be considered exploratory until confirmed through larger, rigorously controlled trials (Dinan et al. 2013; Kinoshita et al. 2021).

Overall, these emerging applications highlight future research opportunities for *L. delbrueckii* , but the current evidence base does not support strong clinical conclusions. Framing these effects as preliminary ensures alignment with the strength of available evidence and underscores the need for targeted mechanistic studies and well‐designed human trials.

## 8 Future Perspectives and Applications of *L. delbrueckii*

The advancement of precision fermentation presents a significant opportunity in the evolution of the dairy industry. This technology enables the targeted use of specific microorganisms, such as *Lactobacillus delbrueckii* , to develop functional dairy products enriched with health‐promoting biomolecules. By employing co‐fermentation strategies—combining starter cultures with probiotics, prebiotics, or bioactive compound‐producing strains—manufacturers can tailor fermentation processes to achieve specific sensory, nutritional, and functional profiles (Teng et al. 2021).

Research into the applications of *L. delbrueckii* in dairy fermentation remains active, driven by its versatile technological traits and multiple health benefits (as discussed in Section 9). Products fermented with *L. delbrueckii* have demonstrated potential for improving gut health, immune function, and other physiological outcomes. Moreover, its application is not limited to human nutrition: supplementing *L. delbrueckii* in livestock operations has been associated with enhanced milk yield and quality, suggesting benefits at the agricultural production level, including improved efficiency and reduced environmental impact (Mo et al. 2022).

*L. delbrueckii* , especially subsp. *bulgaricus* and *lactis*, have long been established in widely consumed dairy products, such as yogurt and cheese. These subspecies have undergone genome streamlining through reductive evolution, optimizing their metabolic pathways for milk‐rich environments. Their genetic profiles influence fermentation dynamics—including acidification rates and metabolic outputs—which can be harnessed to design highly specialized starter cultures (Alexandraki 2020).

Looking forward, future research is expected to focus on unlocking the full metabolic potential of *L. delbrueckii* , particularly in the context of advanced fermentation systems. Precision fermentation may lead to the development of novel dairy products with enhanced nutritional profiles, improved textural and organoleptic characteristics, and targeted functional benefits. Such innovations could redefine traditional dairy production, support the development of entirely new food matrices, and respond to growing consumer demand for sustainable, ethically produced, and health‐oriented foods (Yang et al. 2024). By integrating genomic insights with cutting‐edge food biotechnology, *L. delbrueckii* is poised to play a central role in the next generation of innovative, functional, and sustainable dairy innovation.

### 8.1 Challenges and Research Gaps in the Practical Utility of *L. delbrueckii*

Despite its long history and the accumulation of evidence demonstrating its benefits, the practical, industrial, and clinical use of *L. delbrueckii* presents several challenges that may limit its efficacy in next‐generation functional food systems.

#### 8.1.1 Strain Variability and Lack of Standardization

One significant barrier is the high level of strain‐dependent functional variability. While some strains may exhibit strong acidification, some exopolysaccharide production, and/or some bioactive peptide release, other strains may have different or less desirable functional capabilities in the same matrix. Although this varies by individual, it complicates standardization within the industry and requires significantly more strain characterization before product formulation development (Elean et al. 2023).

#### 8.1.2 Limited Clinical Evidence in Human Trials

There is also a lack of clinical evidence from large‐scale, randomized controlled trials (RCTs) that can provide evidence for the dose‐responsiveness, long‐term safety, and host‐specific efficacy of functional variants of *L. delbrueckii* , particularly for vulnerable populations such as infants, the elderly, and those with metabolic disorders (Abouelela and Helmy 2024).

#### 8.1.3 Stability and Viability in Food Matrices

Maintaining the viability of probiotic strains, such as *L. delbrueckii* , throughout processing, shelf life, and gastrointestinal passage remains a significant technical challenge. Challenges such as sensitivity to O_2_, thermal sensitivity, and acid‐bile sensitivity may affect cell counts prior to reaching the target site in the host (de Jesus, dos Santos Freitas, et al. 2024). Different encapsulation techniques, such as microencapsulation using alginate, whey protein, and resistant starch, may appear viable options, but they are not economically sustainable or scalable in real‐world situations (L. Li et al. 2023).

#### 8.1.4 Regulatory Uncertainty and Strain‐Specific Claims

Most claims for probiotic health will need to be verified by strain‐level testing, as required by the European Food Safety Authority (EFSA) and similar bodies worldwide. Only a handful of strains belonging to *L. delbrueckii* have undergone clinical validation; if a commercial functional food seeks to make claims beyond general nutrition, this presents a considerable regulatory bottleneck (Sanders et al. 2019).

#### 8.1.5 Applicability to Contemporary Food Systems

There are also important implications for contemporary fermented and functional food products, as many fermented foods and functional foods contain multi‐strain or synbiotic combinations, leading to questions surrounding the relationship dynamics (e.g., interference in competition, quorum‐sensing undermining), which may hinder the probiotic nature of probiotic strains. There is a need to explore the dynamics of co‐cultured *L. delbrueckii* and prebiotics, yeasts, or other probiotic strains in different food systems (Yang et al. 2024).

These limitations underscore the need for continued translational research that encompasses strain screening, bioengineering, human trials, and regulatory harmonization to enable the use of *L. delbrueckii* in more reliable and effective non‐dairy probiotic multifunctional products in next‐generation dairy or non‐dairy probiotic products.

## 9 Conclusions

*L. delbrueckii* plays a foundational role in the dairy industry, particularly in the fermentation of yogurt, cheese, and other cultured products. Its well‐established technological functions—including efficient lactose metabolism, proteolytic activity, and exopolysaccharide production—contribute to desirable sensory, textural, and preservative properties in fermented foods. Its rapid acidification and lactic acid production not only ensure fermentation efficiency but also enhance the digestibility of dairy products for individuals with lactose intolerance. Beyond its technological significance, *L. delbrueckii* is increasingly recognized for its probiotic potential, with selected strains demonstrating anti‐inflammatory, immunomodulatory, and metabolic effects mediated by bioactive peptides and other fermentation‐derived metabolites. Together, these attributes position *L. delbrueckii* as a valuable organism for the development of functional foods.

The genetic adaptability of *L. delbrueckii* , particularly its evolution in milk‐rich environments, provides opportunities to design high‐performance strains tailored to specific industrial and nutritional applications. Looking forward, future research should prioritize standardized, strain‐resolved study designs under well‐controlled fermentation conditions and harmonized outcome measures. In particular, multi‐center randomized controlled trials using clearly defined strains, doses, and delivery matrices (e.g., yogurt, cheese, or encapsulated formats) are needed to strengthen clinical evidence and reduce heterogeneity across studies. Parallel omics‐guided characterization combined with mechanistic validation in human‐relevant models would help establish robust links between technological traits and reproducible host‐related effects. Addressing these gaps will improve translational reliability, enable evidence‐based strain selection and regulatory substantiation, and accelerate the development of consistent and targeted functional dairy foods and precision‐fermentation applications.

## Author Contributions

Anahita Barghi, Tara Farhadi, and Mahsa Sadeghi: writing new draft. Yousef Nami and Babak Haghshenas: writing, editing and project administration.

## Funding

The authors have nothing to report.

## Conflicts of Interest

The authors declare no conflicts of interest.

## Data Availability

All data supporting the findings of this study are available within the article.
